# Novel anti-inflammatory effects of the IL-1 receptor in kidney myeloid cells following ischemic AKI

**DOI:** 10.3389/fmolb.2024.1366259

**Published:** 2024-04-17

**Authors:** Yanting Chen, Xiaohan Lu, Raeann L. Whitney, Yu Li, Matthew J. Robson, Randy D. Blakely, Jen-Tsan Chi, Steven D. Crowley, Jamie R. Privratsky

**Affiliations:** ^1^ Center for Perioperative Organ Protection, Department of Anesthesiology, Duke University, Durham, NC, United States; ^2^ Department of Medicine, Duke University, Durham, NC, United States; ^3^ Department of Anesthesiology, Shanxi Province Cancer Hospital, Shanxi Hospital Affiliated to Cancer Hospital, Chinese Academy of Medical Sciences, Cancer Hospital Affiliated to Shanxi Medical University, Shanxi, China; ^4^ Division of Pharmaceutical Sciences, James L. Winkle College of Pharmacy, University of Cincinnati, Cincinnati, OH, United States; ^5^ Neuroscience Graduate Program, University of Cincinnati College of Medicine, Cincinnati, OH, United States; ^6^ Stiles-Nicholson Brain Institute, Florida Atlantic University, Jupiter, FL, United States; ^7^ Department of Microbiology and Molecular Genetics, Duke University, Durham, NC, United States; ^8^ Department of Pharmacology and Cancer Biology, Duke University, Durham, NC, United States; ^9^ Durham VA Medical Center, Durham, NC, United States

**Keywords:** acute kidney injury, myeloid cells, cytokines, interleukin-1, ischemia-reperfusion

## Abstract

**Introduction:** Acute kidney injury (AKI) is one of the most common causes of organ failure in critically ill patients. Following AKI, the canonical pro-inflammatory cytokine interleukin-1β (IL-1β) is released predominantly from activated myeloid cells and binds to the interleukin-1 receptor R1 (IL-1R1) on leukocytes and kidney parenchymal cells. IL-1R1 on kidney tubular cells is known to amplify the immune response and exacerbate AKI. However, the specific role of IL-1R1 on myeloid cells during AKI is poorly understood. The objective of the present study was to elucidate the function of myeloid cell IL-1R1 during AKI. As IL-1R1 is known to signal through the pro-inflammatory Toll-like receptor (TLR)/MyD88 pathway, we hypothesized that myeloid cells expressing IL-1R1 would exacerbate AKI.

**Methods:** IL-1R1 was selectively depleted in CD11c^+^-expressing myeloid cells with *CD11cCre* ^*+*^ */IL-1R1* ^*fl/fl*^ (Myel KO) mice. Myel KO and littermate controls (*CD11cCre* ^*-*^ */IL-1R1* ^*fl/fl*^–Myel WT) were subjected to kidney ischemia/reperfusion (I/R) injury. Kidney injury was assessed by blood urea nitrogen (BUN), serum creatinine and injury marker neutrophil gelatinase-associated lipocalin (NGAL) protein expression. Renal tubular cells (RTC) were co-cultured with CD11c^+^ bone marrow-derived dendritic cells (BMDC) from Myel KO and Myel WT mice.

**Results:** Surprisingly, compared to Myel WT mice, Myel KO mice displayed exaggerated I/R-induced kidney injury, as measured by elevated levels of serum creatinine and BUN, and kidney NGAL protein expression. In support of these findings, *in vitro* co-culture studies showed that RTC co-cultured with Myel KO BMDC (in the presence of IL-1β) exhibited higher mRNA levels of the kidney injury marker NGAL than those co-cultured with Myel WT BMDC. In addition, we observed that IL-1R1 on Myel WT BMDC preferentially augmented the expression of anti-inflammatory cytokine interleukin-1 receptor antagonist (IL-1ra/*Il1rn*), effects that were largely abrogated in Myel KO BMDC. Furthermore, recombinant IL-1Ra could rescue IL-1β-induced tubular cell injury.

**Discussion:** Our findings suggest a novel function of IL-1R1 is to serve as a critical negative feedback regulator of IL-1 signaling in CD11c^+^ myeloid cells to dampen inflammation to limit AKI. Our results lend further support for cell-specific, as opposed to global, targeting of immunomodulatory agents.

## Introduction

Acute kidney injury (AKI) is one of the most common causes of organ failure, occurring in up to 5% of all hospitalized patients (Ronco et al., 2019) and more than 50% of intensive care unit (ICU) patients (Hoste et al., 2015). Several illnesses or exposures can precipitate AKI including sepsis/infection, drug toxicity, and ischemia. Among various etiologies, ischemia-reperfusion injury (IRI) is a frequent clinical complication following transplantation, heart failure, hypovolemia, shock, and vascular clamping during surgical procedures (Ronco et al., 2019). Despite its high morbidity and mortality, there are no effective therapies for AKI aside from supportive care. Thus, there is motivation to identify novel therapeutic targets for AKI.

Kidney myeloid cells are recognized as a critical player in AKI (Han et al., 2019; Xu, 2021). Among kidney myeloid cells, macrophages and dendritic cells play important roles during both the initial injury and subsequent healing process. Unlike in other tissue microenvironments where CD11c (gene name *Itgax*) is used to specifically identify dendritic cells, most non-neutrophil myeloid cells, including macrophages and some monocytes, express CD11c^+^ (Kawakami et al., 2013; Lever et al., 2019; Salei et al., 2020; Zimmerman et al., 2020; Salei et al., 2021; Privratsky et al., 2023). The roles of CD11c-expressing myeloid cells during AKI are complex and context-specific as CD11c^+^ myeloid cells promote cyst formation in a cystic disease model (Zimmerman et al., 2019); whereas deletion of CD11c^+^ cells aggravates cisplatin- and sepsis-induced AKI (Tadagavadi and Reeves, 2010; Privratsky et al., 2023), and impairs the healing process and promotes pro-inflammatory cytokine generation following ischemic AKI (Kim et al., 2010; Lu et al., 2012). Indeed, depending on their activation and subsequent polarization phenotype, CD11c-expressing myeloid cells can secrete both pro- and anti-inflammatory cytokines to modulate the response to kidney injury. The inflammatory pathways that determine myeloid cell phenotype to regulate AKI severity and repair require further elucidation.

During kidney injury, danger signals trigger the release of the canonical pro-inflammatory cytokine interleukin-1 (IL-1)β. IL-1β is produced mainly by activated myeloid cells and binds to its cell surface receptor, IL-1 receptor R1 (IL-1R1), which is typically associated with the activation of pro-inflammatory signaling pathways (Sims and Smith, 2010; Anders, 2016). IL-1R1 has been significantly implicated in AKI as mice with global deletion of IL-1R1 are protected from ischemic and toxic AKI, renal fibrosis following unilateral ureteral obstruction (UUO), and cyst formation (Haq et al., 1998; Jones et al., 2009; Privratsky et al., 2018; Yang et al., 2019). However, when its functions are examined in a cell-specific context, IL-1R1 can serve both protective and detrimental roles (Zhang et al., 2014b; Ren et al., 2022; Ren et al., 2023). One cell type in which the IL-1R1 has been poorly studied for its role in AKI are myeloid cells.

Based on our previous study in a cisplatin AKI model, where we saw reduced inflammatory cytokine expression from IL-1R1-deficient macrophages infiltrating the injured kidneys (Privratsky et al., 2018), we hypothesized that myeloid cells expressing IL-1R1 would exacerbate AKI. To address this hypothesis, we employed a genetic mouse model to specifically delete IL-1R1 from CD11c^+^ myeloid cells and subjected the conditional mutants to IRI AKI. Surprisingly, we found that IL-1R1 on CD11c^+^ cells actually limits ischemic AKI by promoting anti-inflammatory cytokine generation.

## Materials and methods

### Animal experiments

*Animal care.* Male *IL-1R1* ^*fl/fl*^ mice were generously provided by Dr. Randy Blakely (Robson et al., 2016) bred with female *CD11cCre* ^*+*^ mice (Caton et al., 2007) (strain #008068, C57BL/6J congenic background) to obtain *CD11cCre* ^*+*^ */IL-1R1* ^*f/f*^ (Myel KO) mice and *CD11cCre* ^*-*^ */IL-1R1* ^*f/f*^ (Myel WT) littermate control mice. Male mice were randomly assigned to baseline or ischemia-reperfusion (I/R) surgery. Experimenters were blinded to genotype. All of the animal studies were approved by the Durham Veterans Affairs Medical Center (VAMC) Institutional Animal Care and Use Committee, performed at the Durham VAMC, and conducted in accordance with the National Institutes of Health Guide for the Care and Use of Laboratory Animals.

*Ischemia reperfusion model.* Ischemia-reperfusion was performed as previously described via unilateral vascular clamping and contralateral nephrectomy (Suliman et al., 2021). Briefly, mice were anesthetized with ketamine/xylazine. Mice were placed on a warming pad (Hallowell EMC, Pittsfield, MA) heated to 38°C by a Gaymar TP650 water pump. After aseptic prep, a midline dorsal incision was created, and blunt dissection was performed toward right kidney. The flank muscle and fascia above the right kidney was incised and the right kidney was exteriorized after which the renal pedicle was ligated with suture and the right kidney removed. After closure of fascia and muscle over right kidney, blunt dissection was performed toward left kidney. The flank muscle and fascia above the left kidney was incised and the left kidney was exteriorized. Adipose and connective tissue were carefully removed near renal vessels and a 80g pressure clamp (Fine Science Tools, #18055-02) was placed on the left renal pedicle for 26 min. At the end of ischemic time, the clamp was removed, and reperfusion was confirmed by color change in kidney. The fascia and muscle layer and skin were closed by suture and incision infiltrating with 0.25% bupivacaine. The animal was given buprenorphine (0.1mcg/gm) in normal saline subcutaneously for post-operative pain control. The animal was then placed in cage with warming pad during recovery.

### Blood and Serum Analyses

Blood was collected at the indicated time points, allowed to clot for 30 min at room temperature, and centrifuged at 3,000 g for 10 min at 4°C. Serum creatinine levels were measured by LC-mass spectrometry by the University of Alabama at Birmingham (UAB)–University of California San Diego (UCSD) O’Brien Center for Acute Kidney Injury Research Bioanalytical Core (supported by NIH-NIDDK grant DK079337). Serum BUN was measured by kit (Cat# EIABUN, ThermoFisher) according to instructions.

### Histologic injury scoring

Kidney tissues were removed, and a cross-sectional segment obtained. The kidney segment was fixed with 10% neutral-buffered formalin (VWR 16004-128); embedded with paraffin; sectioned in 5 μm sections by the Duke Research Immunohistology Laboratory. Sections were stained with periodic acid-Schiff (PAS) stain and scored by an experienced animal pathologist masked to experimental groups. Sections were graded according to a previously established scoring system (Zhang et al., 2014a): the percentages of tubules with cell lysis/dilation, loss of brush border, and cast formation were scored on a scale from 0-4 (0, no damage; 1, less than 25%; 2, 25%-50%; 3, 50%-75%; 4, >75%). Histologic scores for each kidney were obtained by adding individual component scores.

### RNA extraction and real-time quantitative PCR

RNA was isolated with a Quick-RNATM MiniPrep (Cat# R1055, Zymo Research) or Quick-RNATM MicroPrep (Cat# R1501, Zymo Research) according to the kit instructions. RNA concentration was measured by Nanodrop. The High-Capacity cDNA Reverse Transcription Kit (Cat# 4368813, Invitrogen) was used to synthesize cDNA according to manufacturer’s instructions. Gene expression levels were measured by RT-PCR using Taqman assay (Cat# 4370074, Applied Biosystems) and primers (Supplementary Table S1).

### Western blots

A piece from flash frozen kidneys were homogenized in RIPA Buffer (Cat# R0278, Sigma-Aldrich) containing protease inhibitor (Cat# 1860932, Thermofisher). Protein concentration was measured by PierceTM BCA Protein Assay Kit (Cat# 23227, Thermofisher). 20 μg total protein was loaded into 4%–12% Bis-tris gels and transferred to polyvinylidene difluoride membranes. The membranes were blocked by 5% nonfat milk-TBST for 1 h and then incubated with primary antibodies against NGAL (RRID# AB_355022) or β-actin (RRID# AB_476692) overnight at 4°C. After washing and incubation with secondary antibodies, membranes were then processed with chemiluminescence (Cat# WBLUC0500, Millipore) and detected with Amersham gel imager 680 (Amersham, Buckinghamshire, UK) analysis system. The detected bands were quantified for densitometry by ImageJ.

### Kidney flow cytometry and cell sorting

Kidneys were harvested from naïve or ischemia/reperfusion (I/R) injured Myel WT and Myel KO mice and minced into small pieces, subsequently transferred into a GentleMACS C tube containing 5 mL of digestion buffer (RPMI-1640, 10 μg/mL DNAase, 1 mg/mL Type IV collagenase). The minced kidney was processed using GentleMACS Dissociator. Following mechanical dissociation, the kidney mixture was digested for 30 min at 37°C. Cells were soft spun down at 10 g for 1 min, and supernatant was filtered through a 70 μm cell strainer to remove large cell debris and washed with wash buffer (DPBS, 3% FBS, 2 mM EDTA). Cells were spun down and red blood cells in pellets were lysed with ACK Lysis Buffer (Cat# A10492-01, Gibco), and then the cell suspensions were filter through a 40 μm cell strainer. Cells were spun down, resuspended with Fc-block buffer (Cat# 101320, Biolegend) and incubated for 15 min at 4°C before staining with fluorescently labeled antibodies (Supplementary Table S2), and then live/dead near-IR Dead Cell Stain (Cat# L34976, Invitrogen). Cells were fixed with BD CytoFix (Cat# 554655, BD Biosciences). Prior to analysis, 20 µL of Count Bright absolute counting beads (Cat# C36950, Invitrogen) were added to cells, and samples were acquired on an BD LSRII and analyzed using FlowJo software. The gating strategy was described in Figure 3. Total cell numbers were obtained using enumeration formula as described in manufacturer’s instructions. For cell sorting, digest, blocking, and primary antibody incubation were per above; however, at the end of primary antibody incubation, cells were spun down and resuspended in DPBS + 2% FBS + 2 mM EDTA + 10 μg/mL DNAase + 1/10000 DAPI, and live cells were sorted for populations of interest on an Astrios Sorter (Beckman) by the Duke Cancer Institute Flow Cytometry Core. Cells were collected directly into RNA Lysis Buffer and used for gene expression analysis.

### Immunofluorescence staining

Kidney tissues from *CD11cCre* ^*+*^ mT/mG mice were fixed in 4% paraformaldehyde in PBS for 60 min at room temperature and then transferred to 30% sucrose in DPBS and incubated overnight at 4°C. Samples were embedded in OCT and store at −80°C until use. After sectioning at 5 μm, the cryosections were blocked in 1% BSA for 1 h, followed by DAPI at 1:1000 for 15 min. After washing, the slides were mounted for examination. Images were captured using Axio Imager microscopes (Zesis, Oberkochen, Germany).

#### Cell culture

*Bone marrow derived dendritic cell.* Bone marrow derived dendritic cells (BMDCs) were generated using well established techniques (Hammer et al., 2011; Wagner et al., 2014; Hoste et al., 2015). Briefly, bone marrow was harvested from Myel WT or Myel KO mice by flushing the femurs and tibia with Hank’s Balanced Salt Solution (HBSS) using a 27-gauge needle. Bone marrow cells were cultured in 10 mL of complete RPMI (10% heated-inactivated FBS with 20 mM penicillin/streptomycin) supplemented with GM-CSF from cultured supernatants (kindly provided by Dr. Gianna Hammer, University of Utah) to differentiate them into CD11c^+^ dendritic cells (DCs). On day 3, an additional 10 mL of fresh complete medium containing GM-CSF was added. On day 6, half of the culture medium was replaced with fresh complete medium containing GM-CSF. Upon maturation, typically between day 7 and day 10, cells were more than 90% CD11c^+^ by flow cytometry.

*BMDC stimulation.* Mature BMDCs derived from Myel WT and Myel KO mice were stimulated with vehicle, IL1β (10 ng/mL) or LPS (10 ng/mL) for 12 h. Following treatment, both the cell supernatants and cell lysates were harvested for further analysis.

*HK2 cell.* The human immortalized proximal tubule epithelial cell line HK-2 (ATCC, CRL-2190) was kindly provided by Dr. Tomokazu Souma and cultured in DMEM/F12 supplemented with 10%FBS, 1% penicillin/streptomycin, 1% Insulin-Transferrin-Selenium solution (Cat# 41400-045, Gibco).

*Co-culture BMDCs and HK2 cells.* HK2 cells were seeded into the bottom compartment of 12-well transwell plate (Cat# 76313-904, VWR) a day prior to the initiation of co-culture. Once grown to confluency, HK2 cells were starved for 12 h in FBS-free DMEM/F12 medium. Mature BMDCs derived from Myel WT and Myel KO mice were seeded into insert wells at a density of 0.2 million cells per well and co-cultured with HK2 cells in the presence of vehicle or IL1β for 12 h. Subsequently, HK2 cells were harvested for RNA analysis. For oxygen-glucose deprivation (OGD), HK-2 cells were subjected to 12-h OGD (DMEM no glucose, 92% N2/3% H2/5% CO2) in an anaerobic chamber (Coy Laboratories). After OGD, media was replaced with FBS-free DMEM/F12 medium. Mature BMDCs derived from Myel WT and Myel KO mice were seeded into insert wells at a density of 0.2 million cells per well and co-cultured with HK2 cells for 12 or 18 h in a 37°C growth incubator with 95% air/5% CO2. Subsequently, HK2 cells and BMDC were harvested for RNA analysis.

*Rescue experiment using anakinra.* HK2 cells were grown in 12-well plates to 80%-90% confluence, followed by a 12-h starvation. Cells were then pretreated with anakinra (10 μg/mL) for 1 h prior to exposure to supernatants from either vehicle-treated or IL1β-stimulated BMDCs derived from Myel WT and Myel KO mice for an additional 12 h. Subsequently, HK2 cells were harvested for RNA analysis.

### Single cell RNA sequencing (scRNAseq) analysis

*Kidney digestion and cell isolation.* Our scRNAseq cell isolation protocol has been previously described (Ide et al., 2021; Privratsky et al., 2023). Mice were perfused with ice-cold PBS via cardiac puncture after which the kidneys were harvested. Kidney digest was performed with liberase TM (0.3 mg/mL, Roche, Basel, Switzerland, #291963), hyaluronidase (10 mg/mL, Sigma, H4272), DNaseI (20 mg/mL) at 37 C for 20 min. After centrifugation, cell pellets were incubated with ACK lysing buffer (ThermoFisher #A1049201) and again centrifuged. Pellets were incubated with 0.25%trypsin EDTA at 37 C for 10 min with shaking at 100 rpm in bacterial shaker. Trypsin was inactivated using 10% fetal bovine serum in PBS. Cells were then washed and resuspended in DPBS supplemented with 0.04% bovine serum albumin. After filtration through a 30 μm strainer, cells were loaded onto 10X Chromium System (10X Genomics) at 1000 cells/μl by the Duke Human Vaccine Institute (DHVI) Sequencing Core. The control condition consists of one contralateral, non-ischemic kidney from a wildtype mouse, The ischemic condition consists of one kidney subjected to ischemia-reperfusion surgery. After library preparation, samples were sequenced on a Novaseq 6,000 (Illumina) S2 flow cell by the Duke Center for Genomic and Computational Biology Sequencing Core. Cell Ranger software from 10X Genomics was used to transform raw base call files into FASTQ files (cellranger mkfastq) and align reads with reference mouse genome (cellranger count).

*QC and filtering*. Gene expression matrices generated separately by the *CellRanger* pipeline for each of the two samples were merged together into a single matrix. To exclude low quality cells, we stringently filtered out cells for which fewer than 200 genes/500 unique molecular identifier (UMI)s were detected and excluded likely doublets by removing cells with greater than 150,000 UMIs. We also filtered out cells for which the percent of mitochondrial gene expression represented more than 55% of the total gene expression. These filters removed 4,665 cells. The dataset ultimately consisted of 25702 cells (12,586 for control; 13,116 for ischemia). All genes that were not expressed in at least 1 cell were discarded, leaving 32,285 genes.

*Normalization, sample integration and clustering*. We used the R package *Seurat* v4.3.1 standard workflow for integration of multiple samples to combine the two samples into a unique data set before clustering and visualization. Prior to integration, gene expression was normalized for each sample by scaling by the total number of transcripts, multiplying by 10,000, and then log transforming (log-normalization). We then identified the 2,000 genes that were most variable across each sample. Next, we identified anchor genes between samples using the *FindIntegrationAnchors* function that were used as a basis for *Seurat* to perform the integration of the two samples. We scaled the integrated data before running a Principal Component Analysis (PCA). We then utilized the shared nearest neighbor (SNN) modularity optimization-based clustering algorithm implemented in *Seurat* for identifying clusters of cells. This was performed using the *FindNeighbors* function with 30 PCs, followed by the *FindClusters* function with the Louvain algorithm using a 0.3 resolution. This allowed us to assign cells into a total of 19 clusters. We applied the UMAP method on the cell loadings of the previously selected 30 PCs to visualize the cells in two dimensions.

*Conserved cluster markers and cell identification*. Known markers of cells present in the mouse kidneys were used to manually identify corresponding cell types for each cluster (Park et al., 2018; Clark et al., 2019; Kirita et al., 2020). After annotation and combining of cell subpopulations (i.e., proximal tubule s1, s2, s3 into one PT cluster), the final clustering consisted of 13 different cell populations. Expression of genes of interest were plotted using the DotPlot and FeaturePlot functions within Seurat on the RNA assay.

Macrophage subclustering. A subset of cells corresponding to macrophage clusters was reclustered independently using a similar workflow as for the whole data set.

### Statistical analyses

Figures were generated using Biorender.com. Statistics for scRNAseq analysis were performed as described in the scRNAseq section. Statistical tests and visualization for all other data were performed with GraphPad Prism software. The figures are representative of experiments that were repeated at least twice on different days. An unpaired Student’s t-test with Welch’s correction was used to compare two experimental groups with normal distribution. Comparisons involving more than 2 groups were analyzed by two-way ANOVA with Sidak’s multiple comparisons test for post-hoc analysis or multiple comparisons T-test as appropriate to compare groups. *p* < 0.05 was considered statistically significant.

## Results

### IL-1R1 is expressed at highest levels on CD11c^+^/F4/80^hi^ macrophages

**FIGURE 1:**
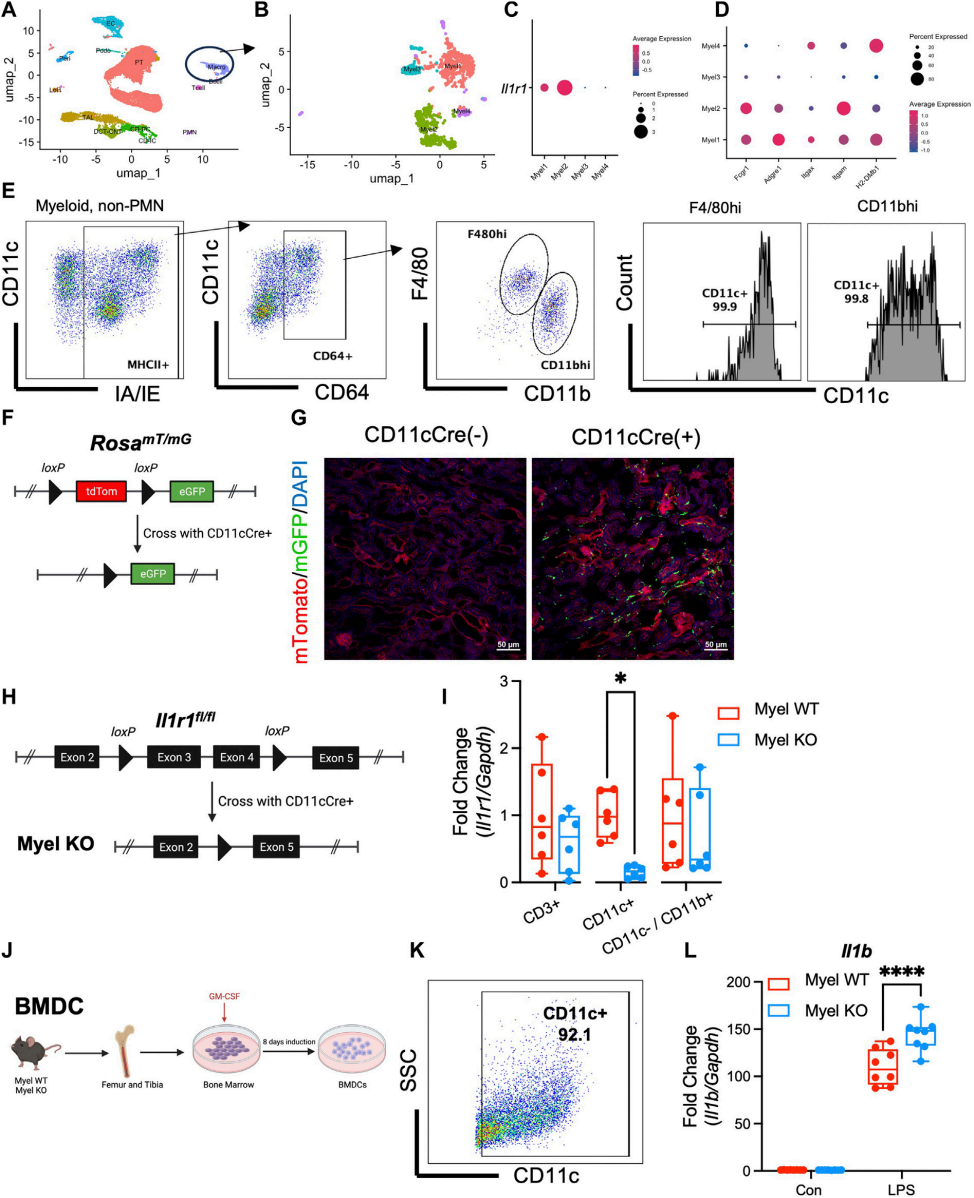
IL-1R1 is expressed in CD11c^+^ myeloid cells and CD11c^+^ IL-1R1 dampens pro-inflammatory cytokine expression. **(A–D)** Wildtype mice were subjected to unilateral ischemia/reperfusion and contralateral uninjured kidney and ischemic kidneys were harvested at 24 h and single cell RNA sequencing was performed. **(A)** UMAP plot of cells clusters in kidneys with “Macro” cluster circled. **(B)** Subclustering of Macro cluster reveals 4 myeloid populations. **(C)** Il1r1 is expressed in two sub-clusters. **(D)** Expression of other common macrophage markers in myeloid sub-clusters. **(E)** Kidneys from uninjured wildtype mice were harvested and subjected to flow cytometry with successive gating strategy shown. Note almost universal CD11c expression in F4/80^hi^ and CD11b^hi^ macrophage populations. **(F)** Genetic strategy for generation of CD11cCre^+^/Rosa^mTmG^ reporter mice (Created with Biorender.com). **(G)** Kidneys from reporter mice were harvested and shown is contour plot is representative immunofluorescence microscopy of ischemic kidneys in CD11c-Cre^+^/Rosa^mTmG^ mice and littermate controls (CD11cCre^−^/Rosa^mTmG^). Note expression of eGFP throughout kidney tissue after I/R. **(H)** Genetic strategy for generating mice with CD11c-specific deletion of IL-1R1 (Myel KO) (Created with Biorender.com). **(I)** Analysis of Il1r1 mRNA expression levels in CD3^+^, CD11c^+^ and CD11c^−^/CD11b^+^ cells sorted from the kidneys of Myel WT and Myel KO mice. Dots represent individual samples from each group (n = 6; *p < 0.05 by multiple comparisons t-test). **(J)** Bone marrow-derived dendritic cells were generated from Myel WT and Myel KO mice (Created with Biorender.com). **(K)** High expression of CD11c in BMDC after 10 days in culture. **(L)** BMDC were stimulated with vehicle (Con) or LPS and RT-PCR was performed for *Il1b* mRNA. (n = 8; ****p < 0.001 by two-way ANOVA with Sidak post-test).

In order to optimally target IL-1R1 deletion to the most relevant intra-renal myeloid cells, we first wanted to identify the intra-renal myeloid cell population(s) on which IL-1R1 is expressed. Since IL-1R1 is known to be expressed at low levels on myeloid cells making it difficult to detect by surface expression (Liu et al., 2019), we performed single cell RNA sequencing (scRNAseq) on control kidneys and clustered cells based on known myeloid cell markers (Figure 1A; Supplementary Figure S1) (Park et al., 2018; Kirita et al., 2020; Privratsky et al., 2023). We further subclustered the myeloid cell cluster into 4 subclusters (Figure 1B) and found that *IL-1R1* is detectable by mRNA expression on two sub-clusters (Figure 1C). We determined mRNA expression of other cell surface markers on subclusters and noted that the Myel1 and Myel2 sub-clusters express cell surface markers similar to F4/80^hi^ and CD11b^hi^ macrophages (Figure 1D). We performed flow cytometry on mouse kidneys and found that F4/80^hi^ and CD11b^hi^ macrophages have near ubiquitous expression of CD11c (Figure 1E). To further determine the expression and localization of CD11c^+^ myeloid cells in the kidney, we crossed the *Rosa* ^*mTmG*^ reporter mouse (Muzumdar et al., 2007) with the *CD11c Cre* mouse line (Caton et al., 2007) (Figure 1F) and performed renal vascular clamping to induce ischemia/reperfusion (I/R) acute kidney injury (AKI). We further noted the presence of CD11c-expressing myeloid cells throughout the kidney (Figure 1G).

### IL-1R1 stimulation on CD11c^+^ myeloid cells suppresses IL-1β expression in vitro

As the predominant IL-1R1-expressing myeloid cells in the kidney also express CD11c, we sought to determine the role of myeloid IL-1R1 signaling in I/R by selectively deleting IL-1R1 from CD11c^+^ myeloid cells in mice. To this end, we crossed an *IL-1R1* ^*fl/fl*^ mouse line (Robson et al., 2016) to the *CD11cCre* line to generate *CD11cCre* ^*+*^ */IL-1R1* ^*fl/fl*^ mice (Myel KO) and littermate control, *CD11cCre* ^*-*^ */IL-1R1* ^*fl/*fl^ mice (Myel WT) (Figure 1H). We isolated intra-renal myeloid cells and detected specific deletion of IL-1R1 in CD11c-expressing cells (Figure 1I). To determine the inflammatory phenotype of CD11c^+^ cells in which IL-1R1 is deleted, we next isolated bone marrow from Myel WT and Myel KO mice and induced CD11c^+^ bone marrow-derived dendritic cell (BMDC)s with GM-CSF (Figure 1J). At 10 days after induction, BMDCs expressed high levels of CD11c (Figure 1K). To induce pro-inflammatory myeloid cell activation, we stimulated BMDC with lipopolysaccharide (LPS). Based on our previous data in cisplatin AKI, where we saw reduced inflammatory cytokine expression in global IL-1R1 KO kidneys (Privratsky et al., 2018), we initially hypothesized that deletion of IL-1R1 in CD11c^+^ BMDC would also limit pro-inflammatory cytokine expression. Contrary to our initial hypothesis, we found that after LPS, BMDC from Myel KO mice expressed higher levels of *Il1b* mRNA compared to BMDC from Myel WT mice (Figure 1L) and upregulated but variable levels of *Tnf* and *Il6* mRNA (Supplementary Figure S2). These data indicate that activation of IL-1R1 in CD11c^+^ myeloid cells might paradoxically constrain inflammation.

### IL-1R1 expression on CD11c^+^ cells attenuates ischemic AKI

**FIGURE 2:**
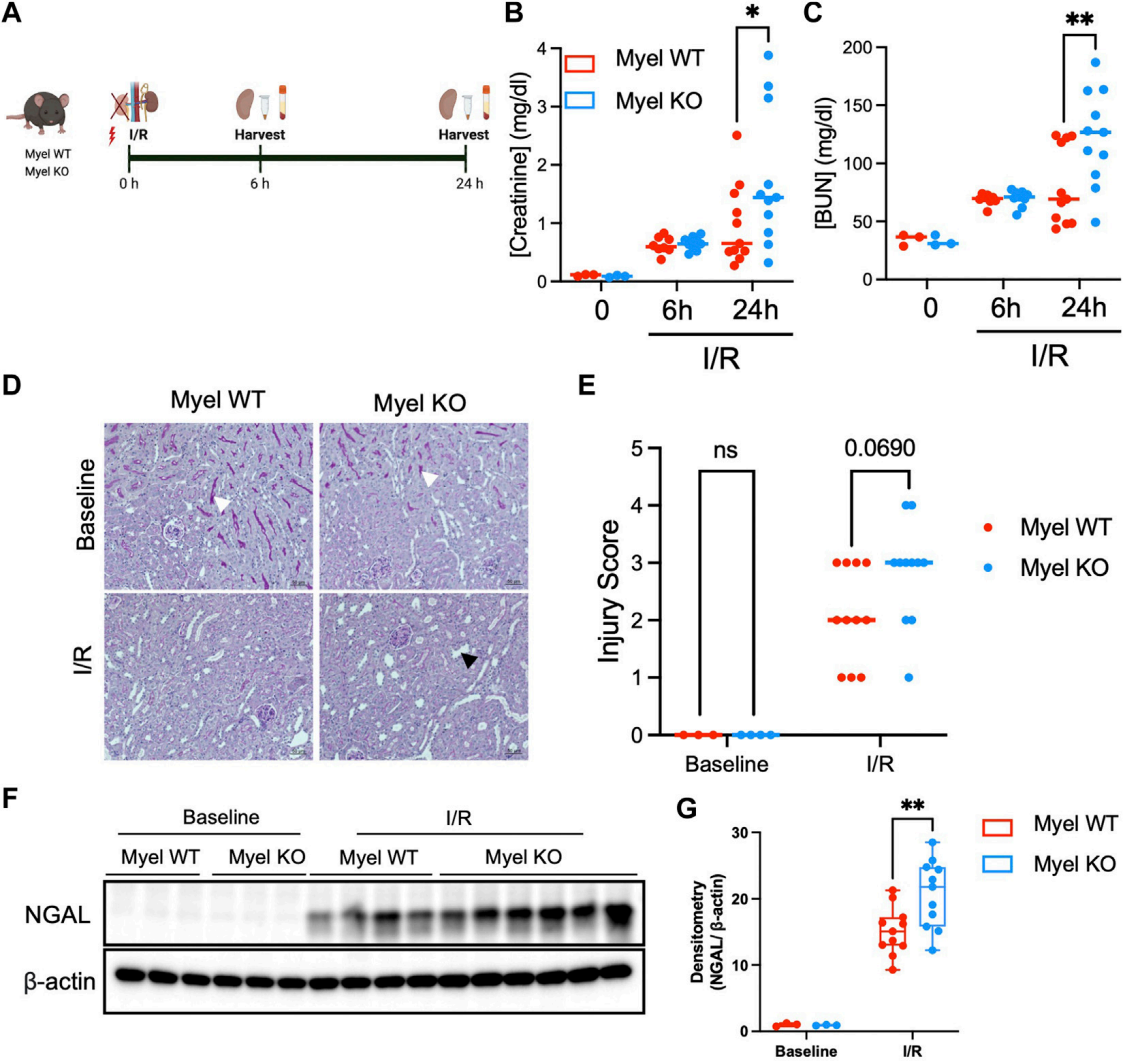
IL-1R1 on CD11c^+^ cells attenuates ischemic AKI. **(A)** Experimental model: Myel WT and Myel KO mice were subjected to ischemia reperfusion surgery for 6 h or 24 h, followed by measurement of (Created with Biorender.com) **(B)** Serum creatinine levels and **(C)** Blood urea nitrogen (BUN) at each indicated timepoints. Dots represent individual samples from each group. Statistical analysis performed by two-way ANOVA with Sidak test. (n = 3 for baseline groups; n = eight to nine mice for 6-h groups; n = 11 for 24-h groups; *p < 0.05, **p < 0.01). **(D)** Representative 20x periodic acid-Schiff (PAS) stained kidney sections from mice subjected to no I/R (Baseline) or 24 h of I/R (scale bar–50 mm, white arrowhead shows brush border in baseline sections, black arrowhead shows example of dilated tubule in I/R sections) with **(E)** quantitation of injury scoring of kidney sections by blinded observer (*p*-value determined by two-way ANOVA). **(F)** Western blot analysis of Neutrophil Gelatinase-Associated Lipocalin (NGAL) in Myel WT and Myel KO kidneys at baseline and 24-h after I/R. **(G)** Densitometry analysis of NGAL normalized to internal control (β-actin). Statistical significance between groups was determined by two way ANOVA with Sidak post-test (**p < 0.01).

To further examine these unexpected findings, we subjected Myel WT and Myel KO mice to kidney ischemia/reperfusion (I/R) surgery and harvested their kidneys at 6 or 24 h after reperfusion (Figure 2A). We noted similar induction of kidney injury biomarkers in Myel WT and Myel KO mice at 6 h; however, compared to Myel WT mice, Myel KO mice demonstrated significantly elevated levels of serum creatinine (Figure 2B) and blood urea nitrogen (BUN) (Figure 2C) at 24 h after I/R injury with a strong trend toward increased injury as measured by histologic injury scoring (Figures 2D, E). To confirm these findings, we further noted that Myel KO mice displayed increased expression of the kidney injury biomarker neutrophil gelatinase-associated lipocalin (NGAL) as measured by Western blot (Figures 2F, G). Taken together, these findings indicated that IL-1R1 on CD11c^+^ myeloid cells limits ischemic AKI.

### IL-1R1 on CD11c^+^ myeloid cells does not modulate intra-renal leukocyte accumulation after ischemic AKI

**FIGURE 3:**
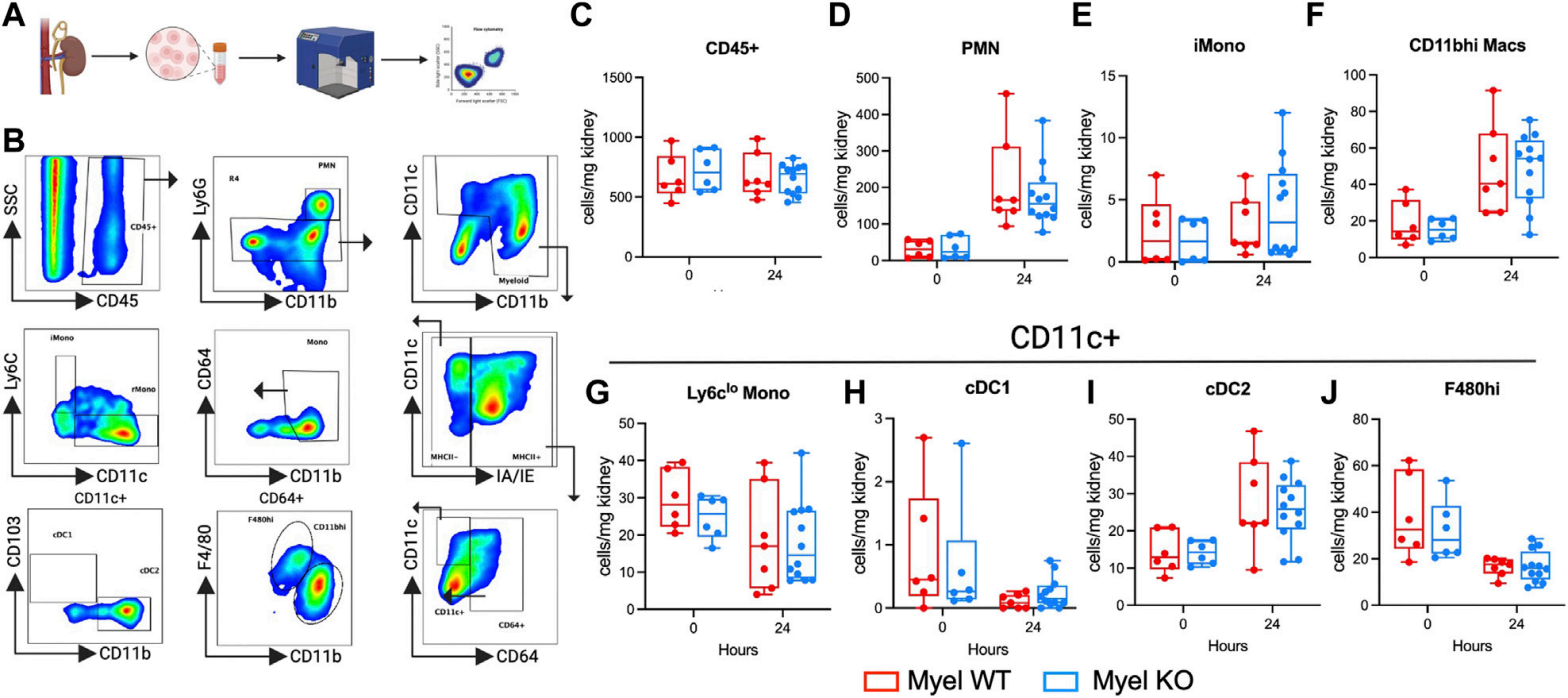
Myel KO mice show similar numbers of leukocyte cell populations compared to Myel WT mice in the kidney at 24-h after I/R. Mice were subjected to sham or I/R, and kidneys were harvested at 24 h. **(A)** Experimental workflow for testing leukocytes accumulations by flow cytometry in kidneys of Myel WT and Myel KO mice (Created with Biorender.com). **(B)** Gating strategy for cell populations. Pseudocolor dot plots for **(C)** CD45^+^, **(D)** neutrophils (PMN), **(E)** Ly6c^hi^ Monocytes (iMono), **(F)** CD11b^hi^ macrophages (CD11b^hi^ Macs). CD11c^+^ populations include **(G)** Ly6c^lo^ Monocytes, **(H)** conventional dendritic cell (cDC)1, **(I)** cDC2, **(J)** F480^hi^ macrophages. Dots represent individual samples from each group. There were no statistically significant differences between genotypes for any cell population as determined by two-way ANOVA with Sidak test.

To identify possible mechanisms for our unexpected findings, we next sought to determine whether IL-1R1 on myeloid cells modulates leukocyte accumulation in the kidney following I/R. We performed flow cytometric analysis on control and ischemic kidneys (Figure 3A). We quantified the proportions of known intra-renal myeloid cell populations (Figure 3B) (Privratsky et al., 2023). We did not detect differences in cell numbers either at baseline or after I/R between Myel WT and Myel KO mice including in CD11c^+^ populations: F480^hi^ macrophages, conventional dendritic cell (cDC)1, cDC2, and Ly6c^lo^ monocytes (Figure 3C). Thus, we concluded that IL-1R1 on CD11c^+^ cells did not significantly modulate their accumulation in the injured kidney.

### Activation of IL-1R1 on CD11c^+^ cells dampens expression of kidney *Il1b* and promotes expression of anti-inflammatory *Il1rn* following I/R injury

**FIGURE 4:**
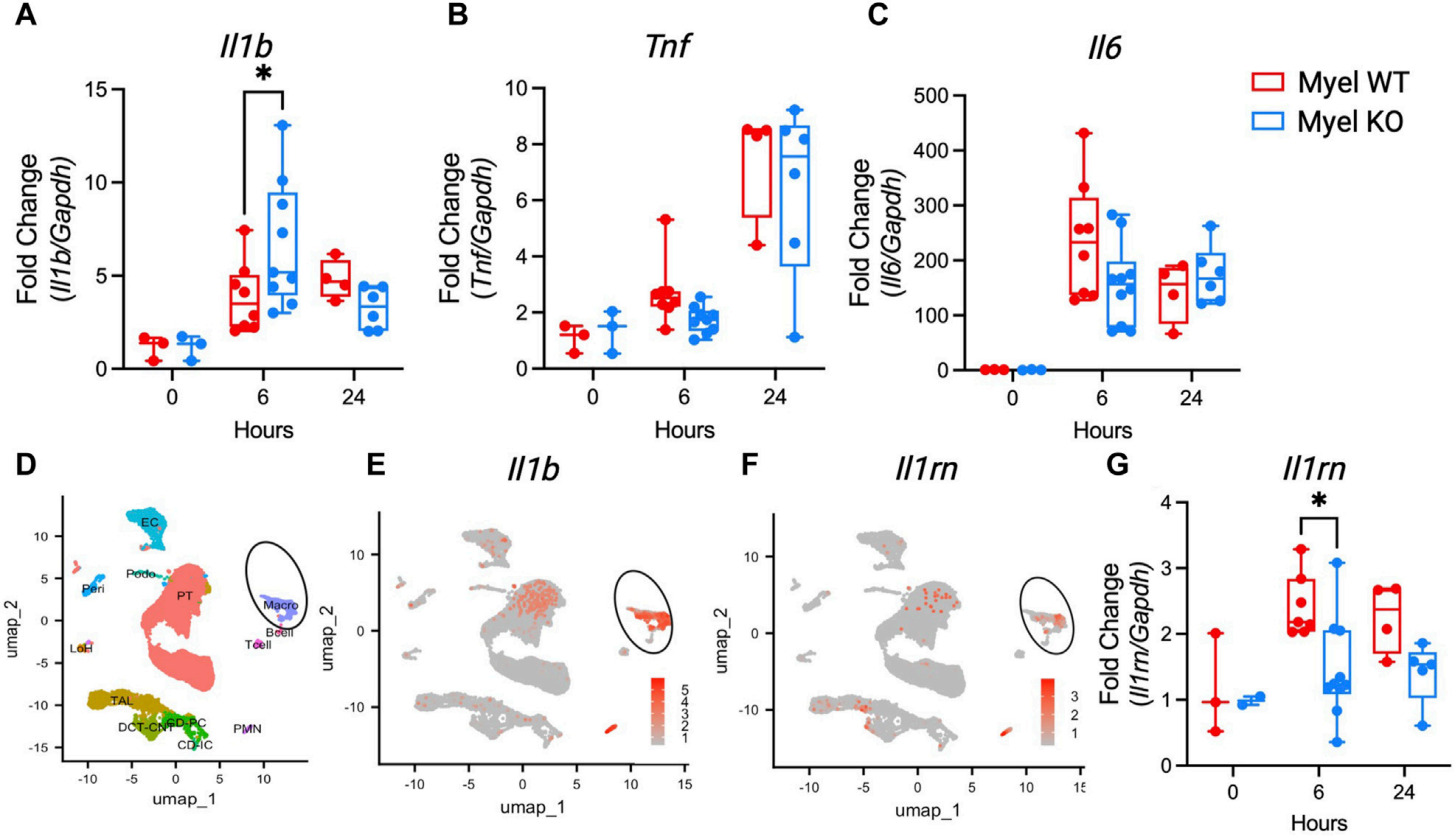
IL-1R1 in CD11c^+^ cells decreases expression of *Il1b* and *Il1rn*. Myel WT and Myel KO mice were subjected to sham or ischemia-reperfusion surgery for 6 h or 24 h. Kidneys were harvested and mRNA levels of **(A)** *Il1b* **(B)** *Tnf* and **(C)** *Il6*. **(D)** UMAP plot of cell clusters from scRNAseq analysis of wildtype mouse kidneys in control and ischemic kidneys. Circle denotes myeloid/macro cluster. **(E)** Feature plots showing *Il1b* expression. **(F)** Feature plots showing *Il1rn* expression. Note major expression of both *Il1b* and *Il1rn* occurs in myeloid cell clusters. **(G)** RT-PCR of anti-inflammatory cytokine *Il1rn* mRNA levels in kidneys from Myel WT and Myel KO mice after sham, 6-h or 24-h I/R surgery. Dots represent individual samples from each group. Statistical analysis performed by two-way ANOVA with Sidak test (n = 3 for sham groups; n = 8-10 for 6-h groups; n = four to six for 24-h groups; *p < 0.05).

As there were minimal changes in kidney leukocyte populations, we next sought to determine whether IL-1R1 on CD11c^+^ cells modulates cytokine generation. We measured cytokine mRNA expression in the kidney after I/R. Similar to our finding in Myel KO BMDC after LPS stimulation, we again found that *Il1b* is paradoxically elevated in Myel KO kidneys compared to Myel WT kidneys at 6 h after ischemic AKI (Figure 4A), with limited effects on expression of other canonical pro-inflammatory cytokines (Figures 4B, C). We were surprised by this finding as myeloid cells were proposed as the primary source of IL-1β (Garlanda et al., 2013), and Myel KO mice lack the receptor for IL-1β. To determine whether myeloid cells are the primary expressors of *Il1b* mRNA, we generated feature plots from scRNAseq data of control and ischemic kidneys from wildtype mice. We confirmed that myeloid cell clusters express *Il1b* at high levels (Figures 4D,E). We additionally found that myeloid cells are major expressors of *Il1rn*/IL-1ra (Figure 4F), an important feedback inhibitor of the IL-1 pathway. Using the Kidney Precision Medicine Project (KPMP) database, we verified that myeloid cells are primary expressors of *IL1RN* in human kidneys following AKI (Supplementary Figure S3). Based on these findings, we hypothesized that IL-1R1 stimulation on myeloid cells could contribute to feedback inhibition of the IL-1 pathway through generation of IL-1ra. In support of our hypothesis, we analyzed whole kidney *Il1rn* gene expression and found that Myel KO mice produce less anti-inflammatory *Il1rn* at 6 h after ischemic AKI compared to WTs (Figure 4G). These results suggest that IL-1R1 on kidney myeloid cells might regulate its own inhibitor, IL-1ra, as a mechanism of kidney protection.

### Activation of IL-1R1 on CD11c^+^ myeloid cells generates IL-1ra to limit IL-1β-induced renal tubular cell injury in vitro

**FIGURE 5:**
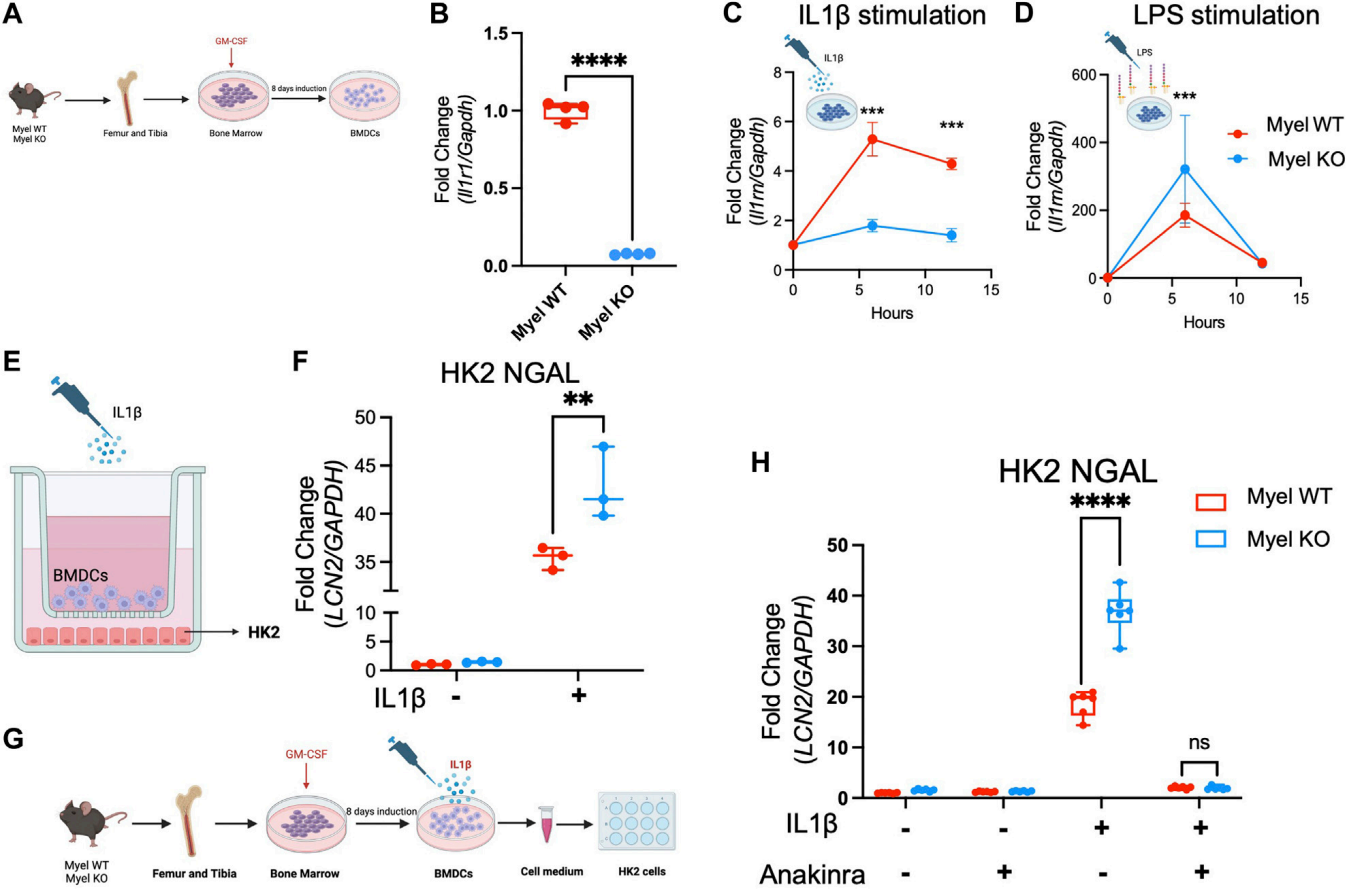
IL-1R1 on CD11c^+^ BMDC generates IL-1ra to limit IL1β-induced renal tubular cell injury *in vitro*. Myel KO GM-CSF-derived bone marrow dendritic cells (BMDCs) produce less anti-inflammatory Il1rn in response to IL1β which induce increased HK2 kidney tubular cellular injury, and IL-1ra (Anakinra) treatment reverses the heightened HK2 cellular injury. **(A)** Schematic experimental workflow for induction of BMDCs from Myel WT and Myel KO mice (Created with Biorender.com). **(B)** Il1r1 mRNA expression in Myel WT and Myel KO BMDC. **(C)** Il1rn mRNA expression in Myel WT and Myel KO BMDCs upon IL1β (Created with Biorender.com) or **(D)** LPS stimulation (Created with Biorender.com) (n = 4; ***p < 0.001). **(E)** HK2 kidney tubular cells were co-cultured with BMDCs from Myel WT or Myel KO mice in transwell system for 12 h (Created with Biorender.com). **(F)** NGAL (*LCN2*) mRNA expression was measured in HK2 cells. Dots represent individual samples from each group. Statistical analysis performed by two-way ANOVA with Sidak test. (n = 3; **p < 0.01). **(G)** Diagram of experimental workflow. HK2 cells were treated with cell medium harvested from either vehicle or IL1β-stimulated Myel WT or Myel KO BMDCs. Media was subsequently treated with vehicle or recombinant IL-1Ra (Anakinra) (Created with Biorender.com. **(H)** Shown in graph is fold change in Ngal mRNA from HK2 cells. Significance was determined by two-way ANOVA with Sidak test (n = 6; ****p < 0.0001).

**FIGURE 6:**
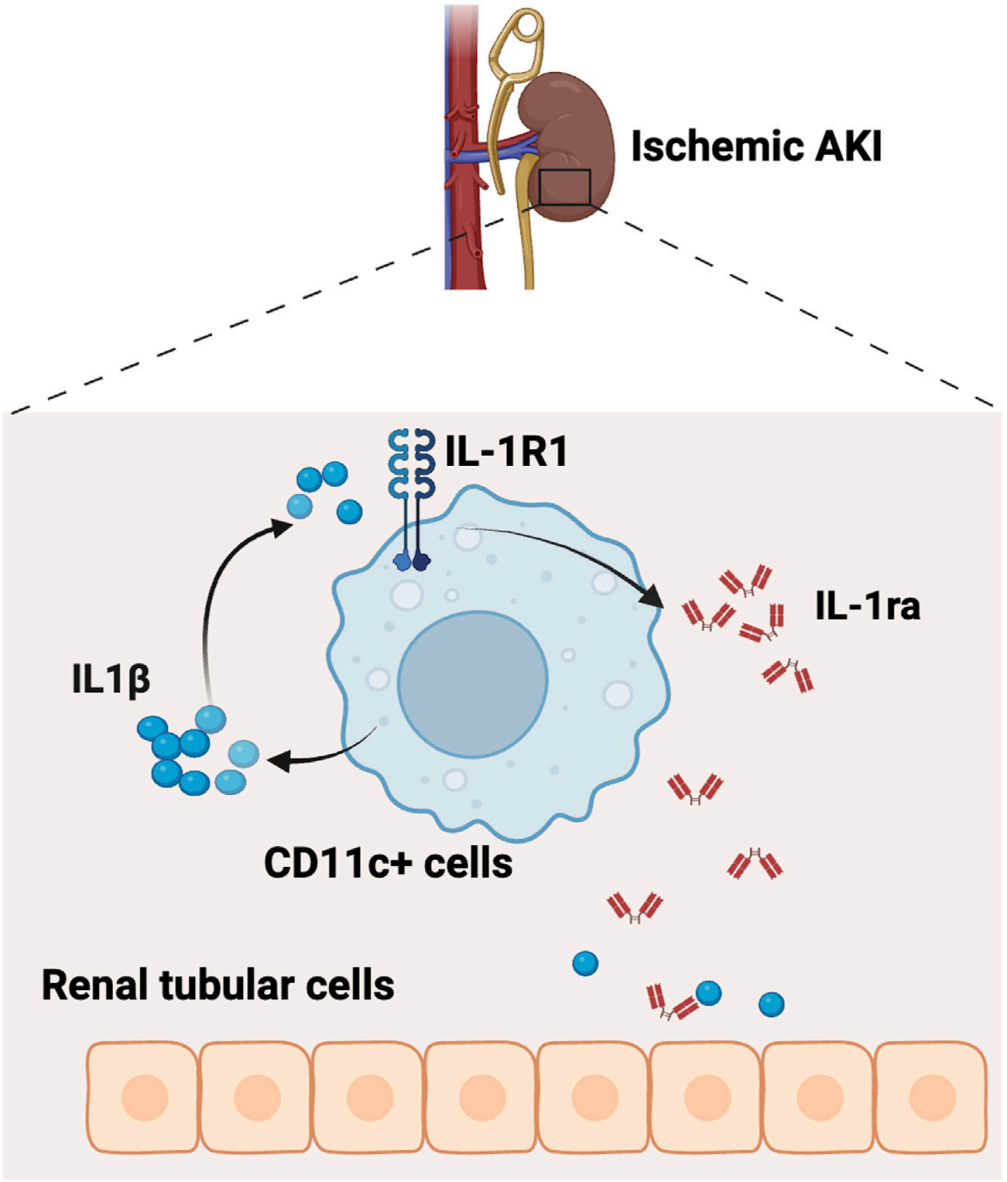
Schematic of working model. The IL-1R1 receptor on CD11c^+^ cells serves as a negative feedback regulator to constrain renal tubular injury by producing anti-inflammatory cytokine IL-1ra during ischemic AKI (Created with Biorender.com).

To further explore whether IL-1R1 signaling in myeloid cells generates IL-1ra to limit kidney tubular injury, we created an *in vitro* co-culture system and tested our hypothesis. We first generated BMDCs from Myel WT and Myel KO mice (Figure 5A). We confirmed deletion of IL-1R1 in BMDCs from Myel KO mice (Figure 5B). We stimulated BMDCs with IL-1β to mimic the sterile inflammation that would be observed in AKI. As expected, we saw reduced expression of *Il1rn* in Myel KO BMDCs compared to Myel WT BMDCs (Figure 5C). We confirmed that Myel KO BMDCs were capable of expressing *Il1rn* using LPS as a positive control (Figure 5D). We next co-cultured Myel WT or Myel KO BMDCs with the renal tubular cell line HK2 in the presence or absence of IL-1β (Figure 5E). Compared to HK2 co-cultured with Myel WT BMDCs, HK2 co-cultured with Myel KO BMDCs showed increased kidney tubular injury as measured by expression of *lipocalin2* that encodes NGAL (Figure 5F). To determine whether IL-1ra is sufficient to ameliorate HK2 injury induced by Myel KO BMDCs, we collected media from IL-1β-stimulated Myel WT and Myel KO BMDCs. We confirmed that compared to media from Myel WT BMDCs, media from Myel KO BMDCs induces increased NGAL expression in HK2 cells (Figure 5H). We then added recombinant IL-1Ra (Anakinra) to restore deficient IL-1ra in Myel KO BMDC media. We found that the addition of recombinant IL-1Ra was sufficient to blunt the increased HK2 injury (Figure 5H). Using oxygen-glucose deprivation (OGD) as an *in vitro* model of I/R, we confirmed that compared to HK-2 cells co-cultured with Myel WT BMDC, Myel KO BMDC express lower levels of *Il1rn* at 12 h after OGD, and higher *Il1b* at 18 h after OGD, which correlates with increased HK-2 cellular injury (Supplementary Figure S4). Taken together, these results suggest that activation of IL-1R1 on CD11c^+^ cells generates an endogenous inhibitor of the IL-1 pathway, IL-1ra, to limit IL-1β-mediated kidney tubular injury (Figure 6).

## Discussion

In the current study, we investigate the role of myeloid IL-1R1 signaling in the pathogenesis of ischemic AKI. As myeloid cells are the primary generators of the pro-inflammatory cytokine IL-1β, we initially hypothesized that deleting its receptor, IL-1R1, in myeloid cells would be beneficial. However, we surprisingly found that IL-1R1 serves as an inhibitory receptor in myeloid cells, curbing inflammation and limiting AKI. Paradoxically, deleting IL-1R1 in CD11c^+^ myeloid cells both increased *Il1b* mRNA expression and reduced the expression of its endogenous inhibitor, *Il1rn*. In support of this inhibitory feedback mechanism, recombinant IL-1Ra treatment was able to reverse the heightened kidney tubular cell injury induced by deletion of IL-1R1 in CD11c^+^ myeloid cells. Thus, our study establishes a protective role for IL-1R1 on CD11c^+^ myeloid cells to prevent aberrant inflammation and limit kidney injury.

The IL-1R1 receptor appears to play a complicated role in kidney injury, especially when its function is interrogated in a cell-specific manner. Numerous studies have established an overall detrimental role of IL-1R1 in kidney injury, as global genetic deletion of IL-1R1 reduces AKI and/or kidney fibrosis following I/R (Haq et al., 1998), unilateral ureteral obstruction (Jones et al., 2009), cyst formation (Yang et al., 2019), and cisplatin AKI (Privratsky et al., 2018). However, when interrogated in a cell-specific manner, it has been shown that IL-1R1 on renal tubular epithelium worsens disease following UUO (Zhang et al., 2014b) and toxic AKI (Ren et al., 2023); whereas IL-1R1 on podocytes (Ren et al., 2022) and endothelial cells (Ren et al., 2023) limits toxic AKI. Our study provides further evidence that IL-1R1 plays a protective role in specific cell types as we found that IL-1R1 limits inflammatory signals in myeloid cells. When taken together, it appears that IL-1R1 plays a consistent injurious function in kidney epithelial cells, whereas it is protective in many other less prominent cell populations in the kidney such as podocytes, endothelial cells, and myeloid cells. Indeed, other groups have shown that IL-1/IL-1R1 signaling can have a protective role in disease (Gonzalez-Navajas et al., 2010; Dmitrieva-Posocco et al., 2019). When taken in a translational context, the cell-specific functions of IL-1 that we and others have observed could partially account for the mixed results on the efficacy of IL-1 antagonism in sepsis in multiple clinical trials in the early 1990s (Fisher et al., 1994a; Fisher et al., 1994b; Opal et al., 1997) and for the side effect profile of IL-1 antagonism even when efficacious (Ridker et al., 2017). Indeed, further investigations into isolating mechanisms to harness the protective, while limiting the detrimental, effects of the IL-1/IL-1R1 pathway activation should lead to future treatments not only for AKI but other inflammatory diseases.

A key role of IL-1R1 is to trigger the activation of innate immune responses (Garlanda et al., 2013). IL-1R1 triggers the generation of chemokines, which can modulate leukocyte migration to sites of injury (Liu et al., 2019). However, we did not find significant effects of the myeloid IL-1R1 to modulate leukocyte accumulation in the injured kidney, including in the main CD11c- and IL-1R1-expressing myeloid populations. One limitation is that our current study cannot rule out an effect on leukocyte migration at later timepoints as macrophages typically tend to migrate 2-3 days after injury. Beyond leukocyte migration, IL-1R1 can activate many inflammatory transcription factors within cells with the most studied being NF-κB (Garlanda et al., 2013). Downstream of both IL-1R1 and multiple Toll-like receptors (TLRs), NF-κB triggers the generation of canonical pro-inflammatory cytokines including IL-1β, TNF, and IL-6 (Garlanda et al., 2013). We surprisingly found that deletion of IL-1R1 in myeloid cells in Myel KO mice leads to selectively increased IL-1β generation after ischemic AKI indicating that IL-1R1 could actually inhibit further pro-inflammatory activation of myeloid cells. Indeed, IL-1 has profound and systemic effects, and unimpeded IL-1-mediated signaling can lead to hyperinflammatory injury. To combat the significant systemic effects of IL-1, cells and tissues produce several endogenous inhibitors of the IL-1 pathway, with the best-known being IL-1ra, encoded by gene *Il1rn* (Dinarello, 2009). One target gene of NF-κB is *Il1rn* (Smith et al., 1994). In our study, we found that myeloid cells are the primary expressors of IL-1ra after AKI in both mice and humans. Thus, it is not surprising that the deletion of IL-1R1 in myeloid cells decreased *Il1rn* expression in ischemic kidneys. In support of our findings here, our group has previously shown that IL-1ra expression in F4/80hi macrophages is critical to curb endothelial IL6 generation and alleviate septic AKI (Privratsky et al., 2023). Taken together, our findings support the concept that a major function of IL-1R1 in myeloid cells is to serve as an autocrine or paracrine negative feedback inhibitor to produce anti-inflammatory IL-1ra and antagonize IL-1 signaling. Future studies will be important to determine how anti-inflammatory transcription factors, such as IL-1ra, are selectively upregulated downstream of IL-1R1.

In summary, our study reveals a surprising anti-inflammatory function of IL-1R1 on CD11c^+^ myeloid cells to attenuate ischemic AKI. Moreover, our findings implicate IL-1R1 on CD11c^+^ myeloid cells as an important negative feedback regulator to generate anti-inflammatory cytokines and thereby curb inflammation. Our study highlights the cell-type specific effects of IL-1R1 that may offer further insights into targeting IL-1/IL-1R1 in kidney disease.

## Data Availability

The original contributions presented in the study are publicly available. This data can be found here: Gene Expression Omnibus (GEO) repository, accession number: GSE263369. Publicly available datasets were analyzed in this study. This data can be found here: https://atlas.kpmp.org/explorer/dataviz. Code used for single cell RNA sequencing analysis can be found at: https://gitlab.oit.duke.edu/jrp43/cd11cil1r_ir_chen.git.
