# Supplementary material for: Novel anti-inflammatory effects of the IL-1 receptor in kidney myeloid cells following ischemic AKI
Source: Front Mol Biosci. 2024 Apr 17;11:1366259. doi: 10.3389/fmolb.2024.1366259 (PMC11061482; doi:10.3389/fmolb.2024.1366259)
Supplement: Supplementary file 1 [file DataSheet1.pdf]

Supplementary Material

Novel anti-inflammatory effects of the IL-1 receptor in kidney myeloid cells following AKI

Yanting Chen, Xiaohan Lu, Raeann L. Whitney, Yu Li, Matthew J. Robson, Randy D. Blakely, Jen-Tsan Ashley Chi, Steven D. Crowley, Jamie R. Privratsky*

*** Correspondence:** Jamie R. Privratsky: jamie.privratsky@duke.edu

Supplementary Tables

Supplementary Table 1: Primers used for RT-PCR analysis

**Gene**	**Accession number/catalog number**
Gapdh	Mm99999915_g1
Il1r1	Mm00434235_m1
Il1rn	Mm00446186_m1
Il1b	Mm00434228_m1
Tnf	Mm00443258_m1
Il6	Mm00446190_m1
GAPDH	Hs02786624_g1
LCN2	Hs00194353_m1

**Supplementary Table 2: Fluorescent cell sorting antibodies**			
**Antibody**	**Company**	**Catalog number, RRID**	**µl/test**
Anti-CD16/CD32	BioLegend	101320, RRID:AB_1574973	1.5
FITC anti-CD11b	BioLegend	101206, RRID:AB_312789	0.25
PerCP/Cy5.5 anti-Ly6G	BioLegend	127616, RRID:AB_1877272	0.25
PE anti-CD11c	BioLegend	117308, RRID:AB_313777	0.25
PE-Cy7 anti-F480	BioLegend	123114, RRID:AB_893490	1
APC anti-CD64	BioLegend	139306, RRID:AB_11219391	1
BV421 anti-CD103	BioLegend	121422, RRID:AB_2562901	1
BV510 anti-Ly6C	BioLegend	128033, RRID:AB_2562351	0.5
BV785 anti-CD45	BioLegend	103149, RRID:AB_2564590	0.1

Supplementary Figures

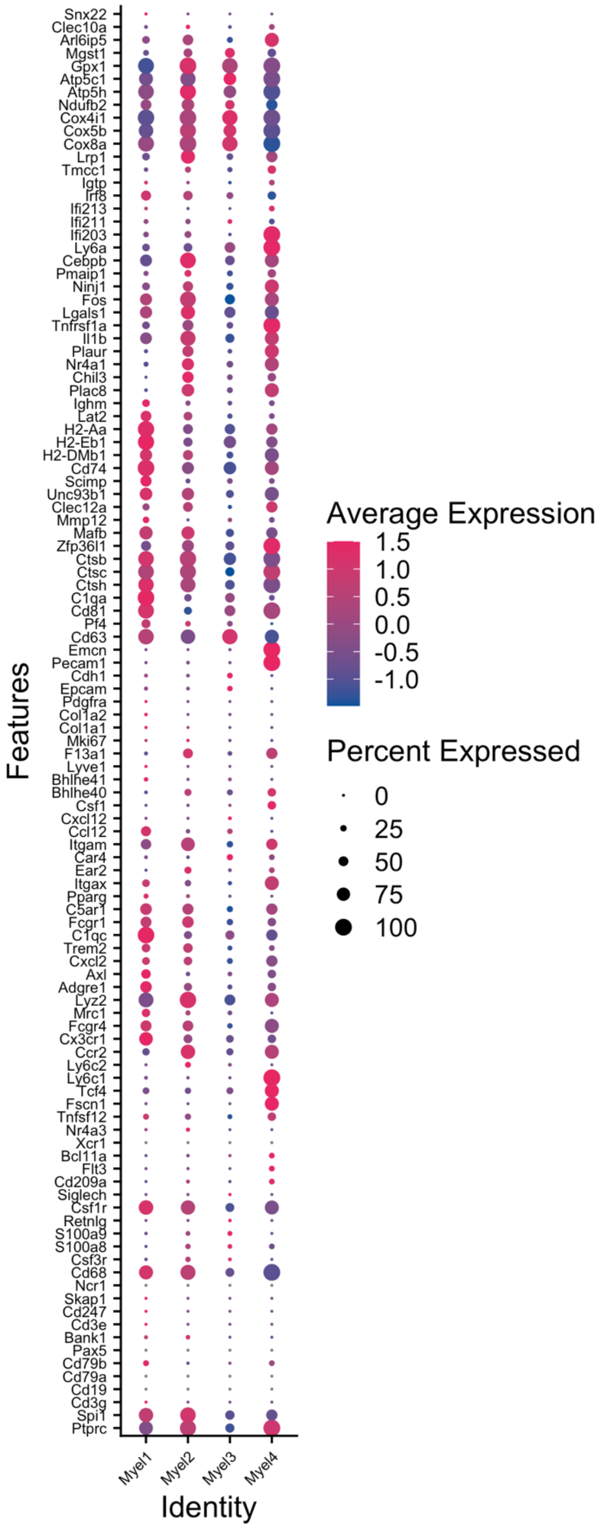

Supplementary Figure S1. Expression of known leukocyte and myeloid cell markers in myeloid sub-clusters by scRNAseq

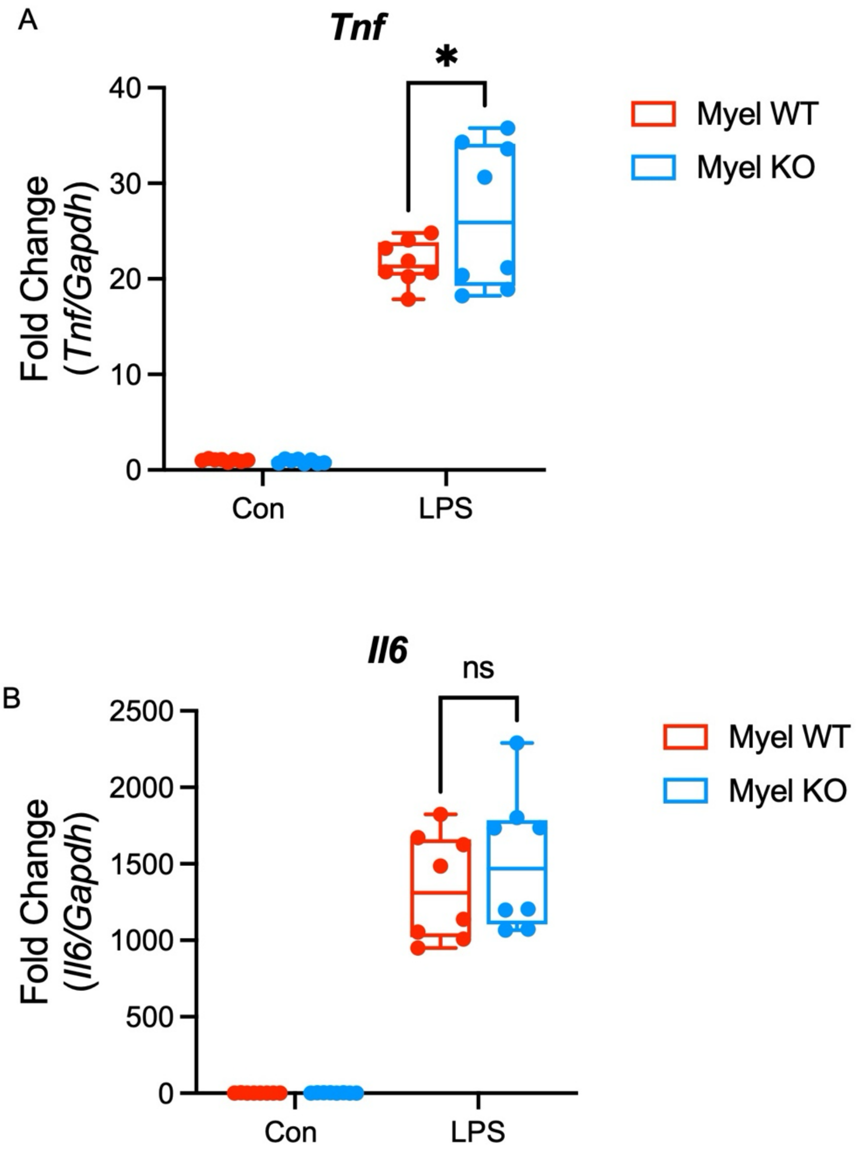

Supplementary Figure S2. The effect of IL1R1 in CD11c+ bone marrow-derived dendritic cells on *Tnf* and *Il6* mRNA expression. Bone marrow-derived dendritic cells were generated from Myel WT and Myel KO mice, were stimulated with Vehicle (Con) or LPS, and RT-PCR was performed for *Tnf* and *Il6* mRNA. (n = 8; *p<0.05 by two-way ANOVA with Sidak post-test).

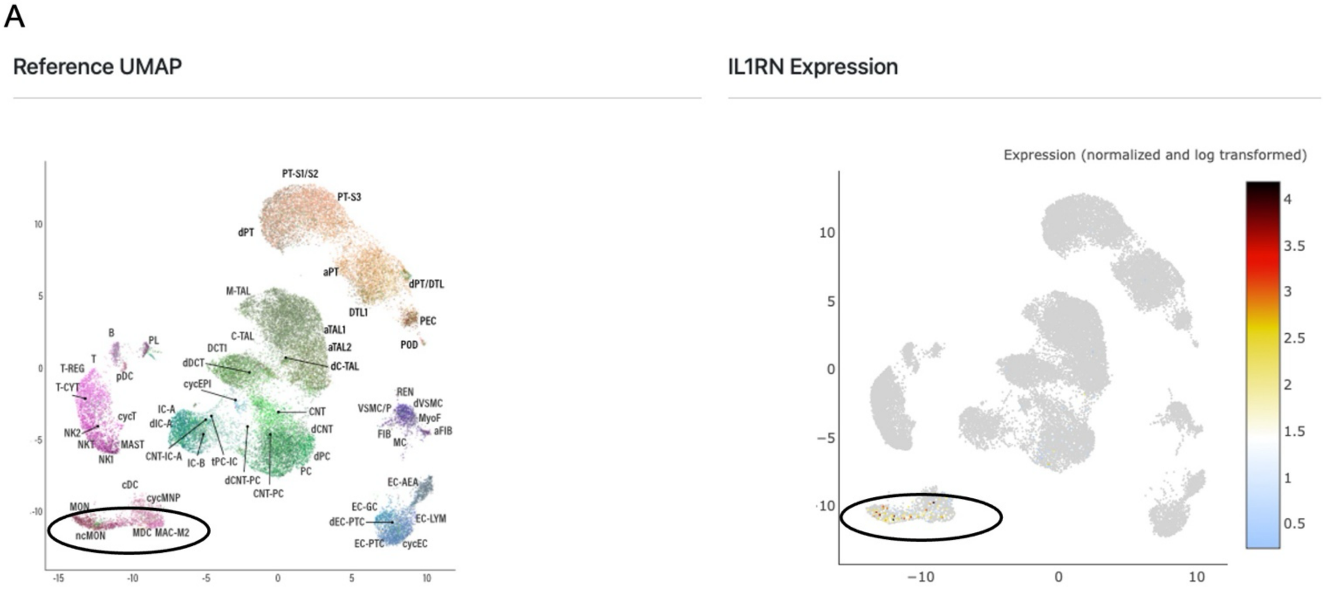

B

Cluster	Cluster Name	Fold Change	Adjusted p val
MAC-M2	M2-Macrophage	0.752	7.94E-38
ncMON	Non-classical Monocyte	1.06	1.79E-30

Supplementary Figure S3. Expression of *IL1RN* in human kidneys following AKI as measured by single cell RNA sequencing. (A) UMAP plot showing cell clusters in database with feature plot for *IL1RN* at right. Non-classical monocytes (ncMON) and M2 Macrophages (MAC-M2) clusters are denoted by black circle. (B) Table showing the cell clusters with significant fold change in *IL1RN* gene expression relative to other cell clusters (https://atlas.kpmp.org/explorer/dataviz).

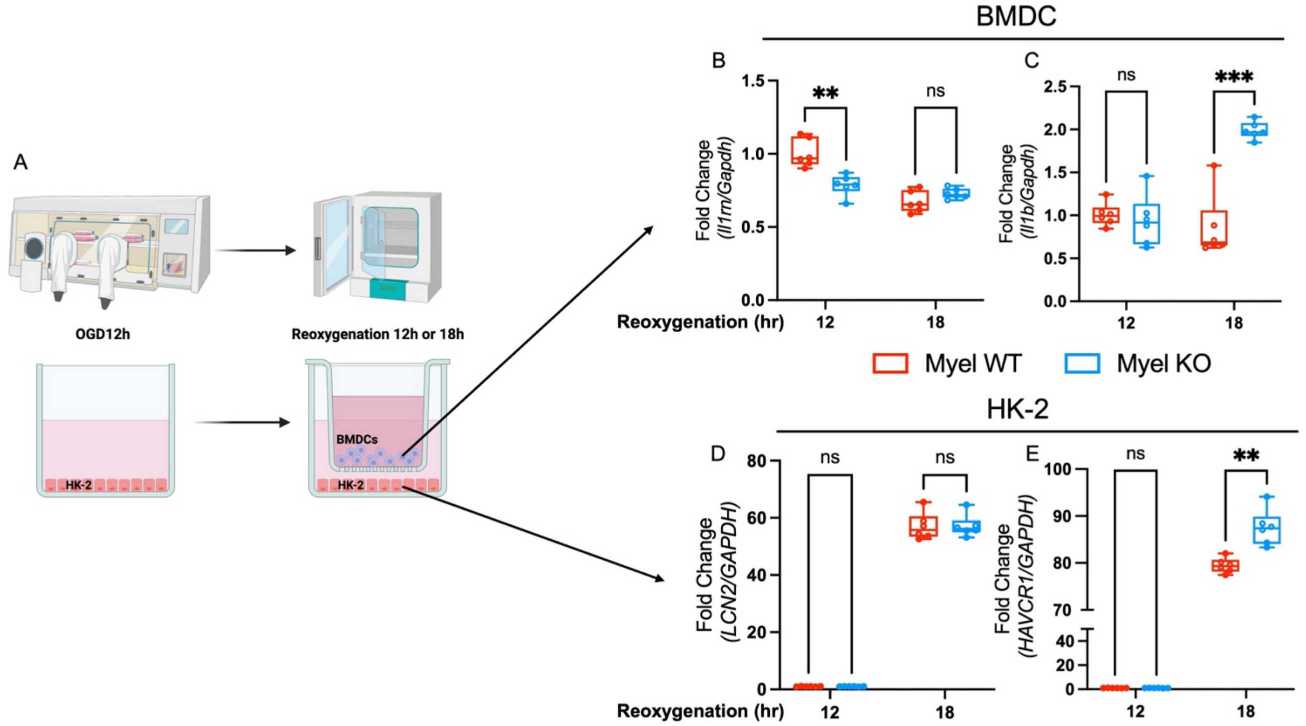

Supplementary Figure S4. IL-1R1 on CD11c$^+$ BMDC limits ischemic renal tubular cell injury in vitro. (A) Schematic experimental workflow for oxygen-glucose deprivation of HK-2 cells and co-culture with BMDC (Created with Biorender.com). (B-E) After OGD and co-culture for indicated timepoints, mRNA was collected from BMDC and HK-2. Shown in graphs are (B) *Il1rn* and (C) Il1b mRNA expression in Myel WT and Myel KO BMDC; and (D) *LCN2*/NGAL and (E) *HAVCR1*/KIM-1 mRNA expression in HK-2 cells (n = 6; *p<0.05 by multiple comparisons T test).
